# Supplementary material for: Drug repurposing for aging research using model organisms
Source: Aging Cell. 2017 Jun 16;16(5):1006–15. doi: 10.1111/acel.12626 (PMC5595691; doi:10.1111/acel.12626)
Supplement: Supplementary file 7 — Data S1 Zip‐Archive of all report cards. [file ACEL-16-1006-s007.zip › RC_CLW.pdf]

CLW

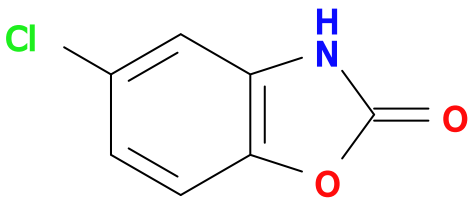

Database identifiers

ChEMBLCompound	CHEMBL1371
DrugBank	DB00356
CHEBI	3655
ZINC	ZINC00897320
eMolecules	536115
eMolecules	901630

Ranking

	Rank	Score
Drosophila	360/697	0.416
C. elegans	NA	NA

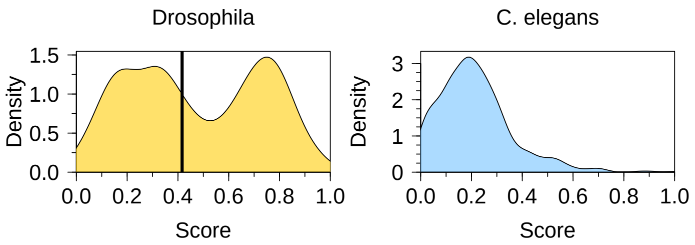

	Ageing implication	Domain conservation	Binding site conservation	Binding affinity	Bioavailability	Lipinski	Promiscuity	Purchasability	Drug approval	**Total**
Drosophila	0.624	0.96	1.0	0.404	(0.9)	0.0	-0.001	0.1	0.1	0.416
C. elegans	NA	NA	NA	NA	NA	NA	NA	NA	NA	NA

Names

- **chlorzoxazone**
- 2-hydroxy-5-chlorobenzoxazole
- 5-chloro-2(3H)-benzoxazolone
- 5-chloro-2-benzoxazolinone
- 5-chloro-2-benzoxazolol
- 5-chloro-2-hydroxybenzoxazole
- chlorzoxane
- Paraflex
- Parafon
- Parafon-Forte
- Strifon

Roles

muscle relaxant, sedative, Intermediate conductance calcium-activated potassium channel protein 4 opener

Status

Approved drug (according to ChEMBL)	Yes
Administration Route	Oral
Number of Rule of 5 violations	0
Binding affinity to original target in log units (RF-Score prediction)	4.61
Burns *C. elegans* bioavailability prediction	4.58

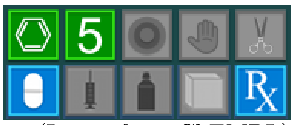

(Image from ChEMBL)

Medical Information

Indicatation: For the relief of discomfort associated with acute painful musculoskeletal conditions.
Mechanism of action: Chlorzoxazone inhibits degranulation of mast cells, subsequently preventing the release of histamine and slow-reacting substance of anaphylaxis (SRS-A), mediators of type I allergic reactions. Chlorzoxazone also may reduce the release of inflammatory leukotrienes. Chlorzoxazone may act by inhibiting calcium and potassium influx which would lead to neuronal inhibition and muscle relaxation. Data available from animal experiments as well as human study indicate that chlorzoxazone acts primarily at the level of the spinal cord and subcortical areas of the brain where it inhibits multisynaptic reflex arcs involved in producing and maintaining skeletal muscle spasm
Toxicity: Oral, mouse: LD50 = 440 mg/kg; Oral, rat: LD50 = 763 mg/kg; Symptoms of overdose include diarrhea, dizziness, drowsiness, headache, light-headedness, nausea, and vomiting.
Metabolism: Chlorzoxazone is rapidly metabolized in the liver and is excreted in the urine, primarily in a conjugated form as the glucuronide.
Retail: A-S Medication Solutions LLC, Barr Pharmaceuticals, Bryant Ranch Prepack, Deliz Pharmaceutical Corp., Direct Dispensing Inc., Dispensing Solutions and 31 more suppliers
Parafon forte dsc 500 mg caplet - 2.65 USD / caplet
Chlorzoxazone 250 mg tablet - 0.18 USD / tablet
Chlorzoxazone 500 mg tablet - 0.87 USD / tablet

(Information from DrugBank)

Compound Target Characteristics

Nitric oxide synthase, endothelial

Best gene implication in ageing for this target family came from gene Q9Z1P9 via mapping the annotation from RGD 3186 annotated in RGD 2014-03-11. Annotation GO 7568 (aging) was Inferred from Expression Pattern

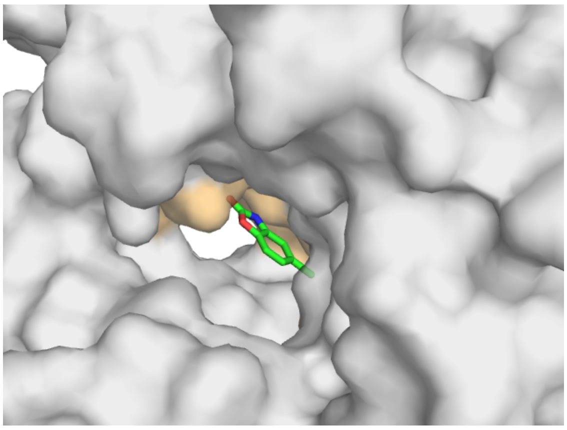

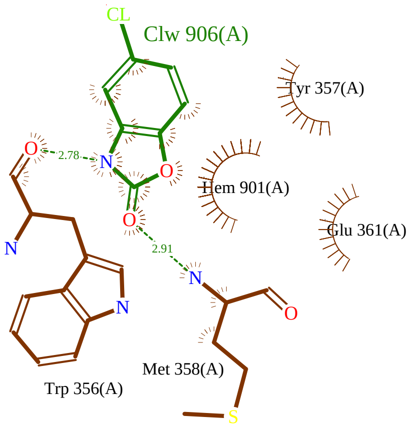

protein	amino acids contacts (binding site)
PDB:1m9j:chainA:P29474	F S G W Y M E
tr:E7ESA7:E7ESA7_HUMAN	F S G W Y M E
sp:P29474:NOS3_HUMAN	F S G W Y M E
tr:Q9Z1P9:Q9Z1P9_RAT	F S G W Y M E
tr:F1LQC7:F1LQC7_RAT	F S G W Y M E
tr:E9Q9X4:E9Q9X4_MOUSE	F S G W Y M E
sp:P70313:NOS3_MOUSE	F S G W Y M E
sp:Q27571:NOS_DROME	F S G W Y M E
sp:P16603:NCPR_YEAST	- - - - - - -

	whole protein		domain-based		contact-based	
protein	ident	simil	ident	simil	ident	simil
PDB:1m9j:chainA:P29474	1.0	1.0	1.0	1.0	1.0	1.0
tr:E7ESA7:E7ESA7_HUMAN	0.83	0.83	1.0	1.0	1.0	1.0
sp:P29474:NOS3_HUMAN	1.0	1.0	1.0	1.0	1.0	1.0
tr:Q9Z1P9:Q9Z1P9_RAT	0.13	0.13	0.76	0.79	1.0	1.0
tr:F1LQC7:F1LQC7_RAT	0.94	0.98	0.96	0.99	1.0	1.0
tr:E9Q9X4:E9Q9X4_MOUSE	0.86	0.9	0.97	0.99	1.0	1.0
sp:P70313:NOS3_MOUSE	0.94	0.98	0.97	0.99	1.0	1.0
sp:Q27571:NOS_DROME	0.4	0.69	0.71	0.92	1.0	1.0
sp:P16603:NCPR_YEAST	0.14	0.37	0.08	0.22	0.0	0.0

Nos (FBgn0011676) associated phenotypes

chemical resistant, developmental rate defective, dominant, eclosion defective, increased cell size, large body, neuroanatomy defective, partially lethal - majority die, small body, some die during pupal stage

(Information from FlyBase)

Nos (UniProt:Q27571) annotation

Function: Produces nitric oxide (NO) which is a messenger molecule with diverse functions throughout the body. Truncated isoforms (isoform 3-isoform 6) are able to form intracellular complexes with the full length protein and serve as dominant negative inhibitors of the enzyme activity. (PubMed:11526108, PubMed:12804606, PubMed:7568075).
Cofactor: heme
Cofactor: FADNote=Binds 1 FAD. ;
Cofactor: FMNNote=Binds 1 FMN. ;
Enzyme regulation: Stimulated by calcium/calmodulin. (PubMed:7568075).
Developmental stage: Isoform 3 is expressed in larvae only. Isoform 4, isoform 5, isoform 6 and isoform 10 are expressed throughout development from embryos to adults. (PubMed:11526108).

(Information from UniProt)

Nitric oxide synthase, inducible

Best gene implication in ageing for this target family came from gene M0RDG2 via mapping the annotation from RGD 3185 annotated in RGD 2014-03-11. Annotation GO 7568 (aging) was Inferred from Expression Pattern

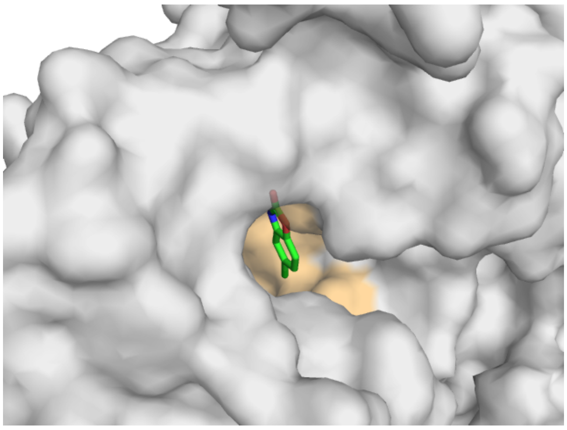

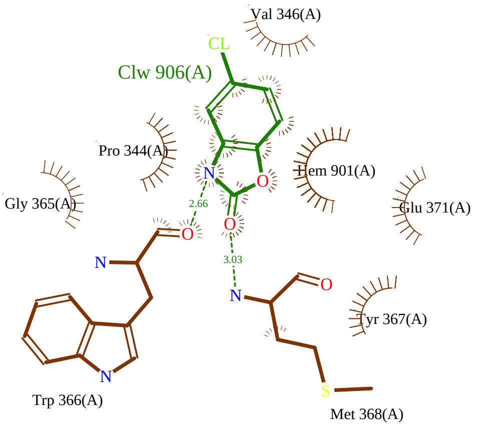

protein	amino acids contacts (binding site)								
PDB:1m8d:chainA:P29477	P	V	F	N	G	W	Y	M	E
sp:P35228:NOS2_HUMAN	P	V	F	N	G	W	Y	M	E
tr:F1LSH9:F1LSH9_RAT	P	V	F	N	G	W	Y	M	E
tr:M0RDG2:M0RDG2_RAT	P	V	F	N	G	W	Y	M	E
sp:P29477:NOS2_MOUSE	P	V	F	N	G	W	Y	M	E
sp:Q27571:NOS_DROME	P	V	F	S	G	W	Y	M	E
sp:P16603:NCPR_YEAST	N	I	D	D	G	D	I	T	S

	whole protein		domain-based		contact-based	
protein	ident	simil	ident	simil	ident	simil
PDB:1m8d:chainA:P29477	1.0	1.0	1.0	1.0	1.0	1.0
sp:P35228:NOS2_HUMAN	0.8	0.93	0.92	0.98	1.0	1.0
tr:F1LSH9:F1LSH9_RAT	0.91	0.95	0.99	1.0	1.0	1.0
tr:M0RDG2:M0RDG2_RAT	0.92	0.95	0.99	1.0	1.0	1.0
sp:P29477:NOS2_MOUSE	1.0	1.0	1.0	1.0	1.0	1.0
sp:Q27571:NOS_DROME	0.38	0.68	0.62	0.91	0.89	0.95
sp:P16603:NCPR_YEAST	0.14	0.38	0.11	0.28	0.11	0.38

Nos (FBgn0011676) associated phenotypes

chemical resistant, developmental rate defective, dominant, eclosion defective, increased cell size, large body, neuroanatomy defective, partially lethal - majority die, small body, some die during pupal stage

(Information from FlyBase)

Nos (UniProt:Q27571) annotation

Function: Produces nitric oxide (NO) which is a messenger molecule with diverse functions throughout the body. Truncated isoforms (isoform 3-isoform 6) are able to form intracellular complexes with the full length protein and serve as dominant negative inhibitors of the enzyme activity. (PubMed:11526108, PubMed:12804606, PubMed:7568075).
Cofactor: heme
Cofactor: FADNote=Binds 1 FAD. ;
Cofactor: FMNNote=Binds 1 FMN. ;
Enzyme regulation: Stimulated by calcium/calmodulin. (PubMed:7568075).
Developmental stage: Isoform 3 is expressed in larvae only. Isoform 4, isoform 5, isoform 6 and isoform 10 are expressed throughout development from embryos to adults. (PubMed:11526108).

(Information from UniProt)
